# Role of preoperative carbohydrate loading: a systematic review

**DOI:** 10.1308/003588414X13824511650614

**Published:** 2014-01

**Authors:** DK Bilku, AR Dennison, TC Hall, MS Metcalfe, G Garcea

**Affiliations:** University Hospitals of Leicester NHS Trust,UK

**Keywords:** Preoperative, Carbohydrate loading, Surgery

## Abstract

**INTRODUCTION:**

Surgical stress in the presence of fasting worsens the catabolic state, causes insulin resistance and may delay recovery. Carbohydrate rich drinks given preoperatively may ameliorate these deleterious effects. A systematic review was undertaken to analyse the effect of preoperative carbohydrate loading on insulin resistance, gastric emptying, gastric acidity, patient wellbeing, immunity and nutrition following surgery.

**METHODS:**

All studies identified through PubMed until September 2011 were included. References were cross-checked to ensure capture of cited pertinent articles.

**RESULTS:**

Overall, 17 randomised controlled trials with a total of 1,445 patients who met the inclusion criteria were identified. Preoperative carbohydrate drinks significantly improved insulin resistance and indices of patient comfort following surgery, especially hunger, thirst, malaise, anxiety and nausea. No definite conclusions could be made regarding preservation of muscle mass. Following ingestion of carbohydrate drinks, no adverse events such as apparent or proven aspiration during or after surgery were reported.

**CONCLUSIONS:**

Administration of oral carbohydrate drinks before surgery is probably safe and may have a positive influence on a wide range of perioperative markers of clinical outcome. Further studies are required to determine its cost effectiveness.

Insulin resistance is a central metabolic change during surgical stress that is directly proportional to the magnitude of the operation. It causes hyperglycaemia in non-diabetic patients. As a consequence, various endocrine and inflammatory systems are stimulated. This results in an exacerbation of the existing postoperative catabolic state with marked loss of body fat and protein stores.^1,2^ Aggressive treatment with insulin to maintain glycaemic control has resulted in reduced organ dysfunction and mortality.^3,4^ Additionally, insulin resistance has been shown to be an independent factor influencing length of stay in hospital postoperatively.^1^

The aim of this review is to systematically appraise the available data regarding the safety and beneficial role of preoperative carbohydrate loading in patients undergoing surgery and, where possible, make comparison with placebo or traditional practice.

## Methods

**Figure 1:**
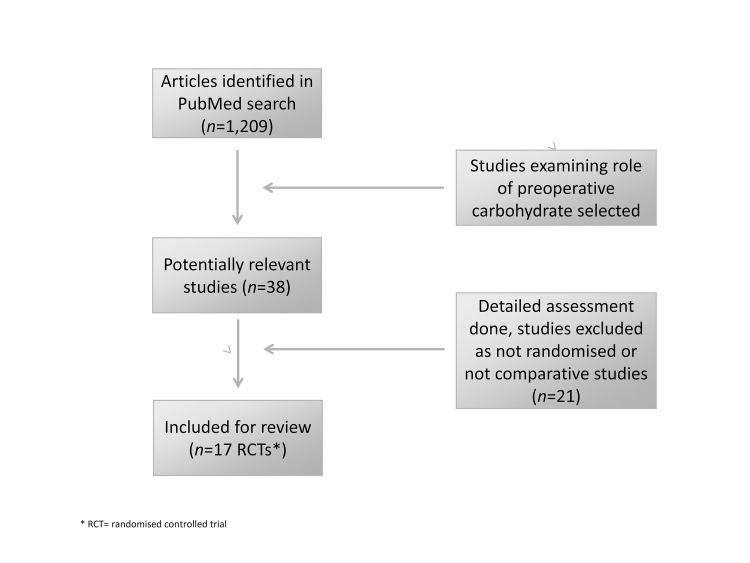
Flow diagram of study selection

A PubMed literature search was undertaken using the keywords ‘carbohydrate loading’, ‘preoperative’, ‘surgery’ and ‘insulin resistance’. Search limits consisted of any article published up until September 2011, studies involving adults undergoing general surgical operations and English language manuscripts. The references of all articles were cross-checked to include all pertinent articles (Fig 1). The primary outcome measure was effect of preoperative carbohydrate loading on insulin resistance. Secondary outcome measures were the effect of carbohydrate treatment on gastric emptying, gastric acidity, wellbeing of patient (assessed qualitatively), immunity, clinical outcome and nutrition.

## Results

Seventeen randomised controlled trials with a total of 1,445 patients who met the inclusion criteria were analysed.^5–21^ The size of the studies varied from 6 to 252 patients. All trials excluded patients with metabolic disorders including diabetes mellitus, ASA (American Society of Anesthesiologists) grade >2, gastro-oesophageal reflux disease and those associated with factors affecting gastric emptying (obesity, pregnancy, sliding hernia of stomach, medications). The protocol for provision of preoperative carbohydrate was variable. Multiple combination of outcomes were analysed by all the studies, making the data too heterogeneous for a meta-analysis.

## Effect of preoperative carbohydrate on insulin resistance

In total, seven articles investigated the effect of preoperative carbohydrate on insulin sensitivity.^5–11^ Various methods were used to analyse insulin resistance (Table 1). Four studies used the hyperinsulinaemic normoglycaemic clamp technique, which is considered to be the gold standard.^8–11^ One article assessed insulin resistance using the HOMA-IR (homeostatic model assessment – insulin resistance) equation.^7^ One study used an artificial pancreas with a closed loop system (STG-22).^5^ One study used the quantitative insulin sensitivity check index.^6^ The time of assessment varied from one week preoperatively to up until three days after surgery. Frequency varied from being assessed once postoperatively or on two separate occasions (preoperative and after surgery).

**Table 1:** Methods used to measure insulin resistance

Technique	Methodology
Hyperinsulinaemic normoglycaemic clamping	This is the gold standard for measuring insulin sensitivity. Insulin is infused intravenously at a rate of 0.8mu/kg/min for 120 minutes. Glucose (200mg/ml) is infused simultaneously, also intravenously, at a variable rate to maintain the blood glucose concentration at 4.5mmol/l. Insulin sensitivity is expressed as the mean glucose infusion rate during a steady-state period during the last 60 minutes.
HOMA-IR	HOMA-IR = insulin (μu/ml) × blood glucose (mg/dl) / 405
Artificial pancreas with a closed loop system (STG-22)	Blood is sampled continuously from a peripheral vein at a rate of 2ml/h and the glucose concentration is monitored. Blood glucose levels are maintained in a target zone by regular, automatic infusion of insulin or glucose into the blood circulation. In the study by Okabayashi *et al*,^5^ the target blood glucose level was set between 80mg/dl and 110mg/dl, and the requirements for insulin to maintain this glucose level for 16 hours following hepatic resection were quantified using the artificial pancreas STG-22.
QUICKI	This is derived from the inverse of the sum of the decimal logarithms of the fasting insulin and fasting glucose, and provides a crude estimation of insulin sensitivity.

HOMA-IR = homeostatic model assessment – insulin resistance; QUICKI = quantitative insulin sensitivity check index

Six trials demonstrated a significant reduction in insulin resistance following the use of preoperative carbohydrate loading (Table 2).^9–11^ The maximum improvement in insulin action observed was by a factor of 50% (*p*<0.01) after the morning dose of carbohydrate on the day of surgery.^12^

**Table 2:** Randomised clinical trials investigating the effect of preoperative carbohydrate on insulin resistance

Study	*n*	Type of surgery	Intervention groups	Technique	Conclusions	*p*-value
Okabayashi, 2010^5^	26	Hepatic resection	1. Control – no additional dietary supplement2. Aminoleban® EN (mixture of carbohydrate and BCAAs) – 50g given orally twice a day started 2 weeks prior to surgery	Artificial pancreas with a closed loop system (STG-22)	IS better in Aminoleban® EN group	**0.039**
Kaska, 2010^6^	221	Colorectal resection	1. Control – overnight fasting2. IV 500ml 10% glucose with 10ml 7.45% KCl and 10ml 20% MgSO_4_ – pm and am3. Oral 400ml potion containing maltodextrin and electrolytes – pm and am	Quantitative insulin sensitivity check index	IS reduced in control group	**0.05**
Faria, 2009^7^	21	Laparoscopic cholecystectomy	1. Overnight fasting2. CHO 200ml – am	HOMA-IR	IS higher in CHO group than fasted group	**0.03**
Svanfeldt, 2007^8^	12	Colorectal resection	1. High CHO group – 125mg/ ml CHO2. Low CHO group – 25mg/ml CHO 800ml – pm, during the waiting period on the day of surgery: 200ml portion given every hour. In total, 3 or 4 portions (600–800ml) ingested, with last portion no later than 2 hours before premedication.	HN clamp – measured before and on the first postoperative day	No effect seen on postoperative peripheral IS	**0.049**
Svanfeldt, 2005^9^	6	Simulated preoperative setting; no surgery	1. Overnight fasting2. CHO 800ml – pm3. CHO 400ml – am4. CHO 800ml – pm, 400ml – am	HN clamp – measured 120 minutes after the morning drink	IS increased by 50% 3 hours after morning drink	**<0.01**
Nygren, 1999^10^	30	Colorectal surgery (*n*=14), THR (*n*=16)	1. CHO 800ml – pm, 400ml – am2. Placebo – similar protocol	HN clamp THR -1 week before surgery and immediately after completion of surgeryColorectal surgery – day before surgery and 24 hours postoperatively	THR: 37% reduction in IS in placebo group immediately after surgery. No significant reduction in IS found in CHO group.Colorectal surgery: 24% greater reduction in IS in fasted group than in CHO group at 24 hours after surgery	**<0.05**
Ljungqvist, 1994^11^	12	Laparoscopiccholecystectomy	1. Control – overnight fasting2. Overnight glucose infusion 5mg/kg/min	HN clamp – measured 3 days preoperatively and on first day postoperatively	IS reduced in control patients compared with treatment group	**<0.01**

BCAAs = branched chain amino acids; IS = insulin sensitivity; pm = evening before surgery; am = morning of surgery; CHO = carbohydrate drink; HOMA-IR = homeostatic model assessment – insulin resistance; HN = hyperinsulinaemic normoglycaemic; THR = total hip replacement

In contrast, only one study demonstrated no effect of carbohydrate on postoperative peripheral insulin sensitivity (borderline significance, *p*=0.049). This may be due to a type II error, the small sample size (12 patients) or the timing of surgery and diverse fasting durations.^8^

### Effect of preoperative carbohydrate on gastric emptying

Five articles investigated the effect of carbohydrate administered preoperatively on gastric emptying (Table 3).^6,12–15^ The protocol used in the trials varied with comparisons made between oral carbohydrate drinks and fasting from midnight or water or oral nutritional supplement or mixture of carbohydrate and soy protein or intravenous glucose and electrolytes. The time and number of drinks administered was also variable.

**Table 3:** Randomised clinical trials investigating the effect of preoperative carbohydrate on gastric emptying

Study	*n*	Type of surgery	Intervention groups	Analysis	Gastric emptying	*p*-value
Kaska, 2010^6^	221	Colorectal resection	1. Overnight fasting2. IV 500ml 10% glucose with 10ml 7.45% KCl and 10ml 20% MgSO_4_ – pm and am 3. Oral 400ml potion containing maltodextrin and electrolytes – pm and am	NG tube	GFV lower in group 3 than in group 1	Not stated
Nygren, 1995^12^	12	Laparoscopic cholecystectomy, parathyroid surgery	1. CHO – 400ml2. Water – 400ml3. Control – protocol repeated among the same patients 53 ±7 days after operation4. The same protocol was performed among healthy volunteers after ingestion of CHO or water.	Gamma cameras and a radiotracer mixed with the drink	No difference. For CHO group: 90 minutes.	**<0.05**
Yagci, 2008^13^	70	Laparoscopic cholecystectomy, thyroidectomy	1. CHO – 800ml pm, 400ml am2. Control – overnight fasting	NG tube	No difference	0.61
Henriksen, 2003^14^	29	Bowel resection	1. CHO – 400ml pm, 400ml am2. CHO + peptide (drink made of 12.5g/100ml carbohydrate and 3.5g/100ml hydrolysed soy protein) – same protocol3. Control – water until 3 hours before induction	Dye dilution technique	No difference. For CHO group: <90 minutes.	Not stated
Hausel, 2001^15^	252	Laparoscopic cholecystectomy, colorectal resection	1. CHO – 800ml pm, 400ml am2. Placebo – same protocol3. Overnight fasting	In 245 patients: NG tubeIn 142 patients: single marker dilution technique	No difference.7 of 245 patients had GFV of >100ml.	Not stated

IV = Intravenous; pm = evening before surgery; am = morning of surgery; NG = nasogastric; GFV = gastric fluid volume; CHO = carbohydrate drink

All the studies reported no difference in gastric emptying times between the groups that received placebo or fasting from midnight or intravenous glucose and carbohydrate drinks.^6,12–15^ However, three patients in the study conducted by Hausel *et al* had large residual gastric fluid volumes.^15^ It was noted that one of these patients had a history of previous intestinal obstruction, one had a short interval between intake of drink and premedication, and the third patient had abnormal fasting plasma glucose.

### Effect of preoperative carbohydrate on gastric acidity

Three randomised trials examined the effect of carbohydrate drinks given preoperatively on gastric acidity in patients undergoing surgery.^6,13,15^ Modes of assessing gastric acidity varied between the studies (Table 4). The study by Hausel *et al* assessed acidity by automatic back titration with sodium hydroxide to pH 7.^15^ A study by Yagci *et al* used a urine pH meter.^13^ The third trial used biochemical indicator paper.^6^ All the studies demonstrated that there was no difference in gastric acidity following a carbohydrate drink compared with placebo or intravenous glucose or, more importantly, fasting.

**Table 4:** Randomised clinical trials investigating the effect of preoperative carbohydrate on gastric acidity

Study	*n*	Type of surgery	Intervention groups	Technique	Conclusions
Kaska, 2010^6^	221	Colorectal resection	1. Overnight fasting2. IV 500ml 10% glucose with 10ml 7.45% KCl and 10ml 20% MgSO_4_ twice – pm and am3. Oral 400ml potion containing maltodextrin and electrolytes – pm and am	Biochemical indicator paper	Gastric pH was comparable for all three groups
Yagci, 2008^13^	70	Laparoscopic cholecystectomy, thyroidectomy	1. CHO – 800ml pm, 400ml am2. Control – overnight fasting	Urine pH meter	Gastric pH was comparable for both groups
Hausel, 2001^15^	252	Laparoscopic cholecystectomy, colorectal resection	1. CHO – 800ml pm, 400ml am 2. Placebo – same protocol3. Overnight fasting	Automatic back titration with sodium hydroxide to pH 7	Gastric pH was comparable for all three groups

IV = intravenous; pm = evening before surgery; am = morning of surgery; CHO = carbohydrate drink;

### Effect of preoperative carbohydrate on patient wellbeing

Eight studies examined the impact of preoperative carbohydrate drinks on patient wellbeing (Table 5).^6,12,14–19^ Six trials used a visual analogue scale (VAS) to assess patient comfort.^12,14–17,19^ The variables measured by the VAS were thirst, hunger, anxiety, depression, pain, tiredness, weakness, inability to concentrate, mouth dryness and nausea. The numbers of variables studied in each trial were different. In the study by Kaska *et al*, the psychosomatic status of patients was assessed by the modified Beck questionnaire,^6^ which consists of 21 questions addressing symptoms such as fatigue and irritability. Hausel *et al* used two methods: VAS and objective analysis by nursing staff.^18^

**Table 5:** Randomised clinical trials investigating the effect of preoperative carbohydrate on wellbeing of the patient

Study	*n*	Type of surgery	Intervention groups	Technique	Conclusions
Kaska, 2010^11^	221	Colorectal surgery	1. Overnight fasting2. IV 500ml 10% glucose with 10ml 7.45% KCl and 10ml 20% MgSO_4_ – pm and am3. CHO 400ml – pm and am	Modified Beck questionnaire	Group 3: Reduced thirst, hunger, anxiety and pain
Nygren, 1995^12^	12	Laparoscopic cholecystectomy, parathyroid surgery	1. CHO 400ml – am2. Water 400ml – until 4 hours before induction of anaesthesia3. Control – protocol repeated among the same patients 53 ±7 days after operation The same protocol was also performed among healthy volunteers after ingestion of CHO or water.	VAS	Thirst was reduced during the first 60 minutes after CHO and 40 minutes after water. Thereafter, no significant changes observed. Hunger was reduced after 20 minutes of water but not after CHO. Anxiety was reduced after water but not after CHO.
Henriksen 2003^14^	, 48	Bowel resections	1. CHO 400ml – pm, 400ml – am2. CHO + peptide (drink made of 12.5g/100ml carbohydrate and 3.5g/100ml hydrolysed soy protein – same protocol3. Control – water until 3 hours before induction of anaesthesia	VAS	No difference found between the groups in thirst, hunger, anxiety, wellbeing, fatigue, pain (pain at rest, with cough and mobilisation) and nausea
Hausel, 2001^15^	252	Laparoscopic cholecystectomy, colorectal surgery	1. CHO 800ml – pm, 400ml – am2. Placebo – similar protocol3. Overnight fasting	VAS	Group 1: Reduced hunger, thirst, anxiety, malaise and unfitnessGroup 2: Increased nausea, tiredness, inability to concentrate. No consistent trend for hunger or thirst.Group 3: Increased hunger, thirst, tiredness, weakness and inability to concentrate
Mathur, 2010^16^	142	Colorectal surgery, hepatic resection	1. CHO 800ml – pm, 400ml – am2. Placebo – similar protocol	VAS	No benefit of CHO demonstrated on anxiety, depression, hunger, thirst, inability to concentrate, malaise, nausea, pain at rest, pain with cough, unfitness or irritability
Helminen 2009^17^	210	Abdominal surgery, thyroidectomy, parathyroid surgery	1. IV 1,000ml 5% dextrose between midnight and 6am2. CHO 400ml – am3. Overnight fasting	VAS	Group 1: Increased thirst, mouth dryness and anxiety. No consistent trend for hunger, weakness or tiredness.Group 2: Reduced thirst. Hunger better than IV glucose group.Group 3: Increased thirst, hunger, tiredness, anxiety, weakness and mouth dryness
Hausel, 2005^18^	172	Laparoscopic cholecystectomy	1. CHO 800m – pm, 400ml – am2. Placebo – similar protocol3. Overnight fasting	Two methods:1) Objective analysis of nausea and vomiting by nursing staff2) VAS	Incidence of nausea and vomiting was similar in the three groups during the first 12 hours. Between 12 and 24 hours, more patients in the fasted group experienced nausea and vomiting than in the CHO group.
Bisgaard, 2004^19^	94	Laparoscopic cholecystectomy	1. CHO 800ml – pm, 400ml – am2. Placebo – similar protocol	VAS	Preoperative CHO had no influence on postoperative discomfort in terms of general wellbeing, fatigue, appetite, pain, nausea, vomiting, sleep and physical activity compared with placebo.

IV = intravenous; pm = evening before surgery; am = morning of surgery; CHO = carbohydrate drink; VAS = visual analogue scale

Preparation with carbohydrate led to a significant reduction in thirst, hunger, anxiety and malaise in two trials compared with fasting and placebo (flavoured water).^15,18^ The improvement in thirst was similar for the placebo and carbohydrate groups. Two trials compared intravenous glucose with fasting from midnight and oral carbohydrate drinks.^6,17^ These studies demonstrated that the fasted patients had increased thirst, hunger, tiredness, anxiety and mouth dryness scores. On the contrary, both intravenous and oral carbohydrates alleviated feelings of tiredness and weakness compared with fasting. However, intravenous glucose infusion did not decrease the sense of thirst and hunger as effectively as oral carbohydrates.

Hausel *et al* investigated the effect of carbohydrate on postoperative nausea and vomiting in 172 patients undergoing an elective laparoscopic cholecystectomy.^18^ Between 12 and 24 hours after surgery, there was a significantly lower incidence of nausea and vomiting in the carbohydrate group than in the fasted group. Three studies demonstrated no beneficial effect of carbohydrate drinks on similar variables measuring general wellbeing of the patient prior to surgery.^14,16,19^

### Effect of preoperative carbohydrate on immunity and clinical outcome

Two trials examined the impact of carbohydrate drinks on postoperative immunity and clinical outcomes.^16,20^ Mathur *et al* conducted the largest double blind placebo controlled trial in 2009 to study the effect of preoperative carbohydrate drinks on a number of clinical outcomes after colorectal surgery and hepatic resection.^16^ There was no difference in the incidence of postoperative infections in the carbohydrate group compared with the placebo group. Furthermore, no significant benefit was observed in the carbohydrate group with regard to length of stay and time to intake of oral diet.

In contrast to this, Noblett *et al* demonstrated that preoperative treatment with carbohydrate drinks reduced the length of hospital stay compared with placebo or water.^20^ Earlier return of gut function was also noticed in the carbohydrate group although this was not statistically significant. The early return of gut function could be a contributory factor for reduced postoperative hospital stay.

### Effect of preoperative carbohydrate on nutrition

Five studies examined the effect of preoperative carbohydrate on the postoperative nutritional status of the patient.^6,14,16,20,21^ Varied methods were employed for measurement of nutrition. Four trials used anthropometric measurements.^14,16,20,21^ A dynamometer measured grip strength in the dominant hand. Other measurements included triceps skinfold thickness and mid-arm circumference. One study measured muscle power in hand grip with a digital tension meter.^6^

In the study conducted by Henriksen *et al*, no significant difference was observed between the groups when analysed per se.^14^ However, when the results of the two intervention groups (carbohydrate only, and carbohydrate and peptide) were pooled together, they had a significantly better muscle strength in the quadriceps muscles than the control (water) group after one month (*p*<0.05). Despite this, no difference was observed between the three groups in voluntary isometric hand grip strength. Noblett *et al* demonstrated a significant reduction in grip strength on discharge in the fasted patients compared with their preoperative values (*p*<0.05).^20^ In contrast, both the carbohydrate and placebo groups did not show a significant reduction in their postoperative grip strength. Similar results were noted by Kaska *et al* but the values were not significant.^6^

Yuill *et al* found no significant difference in the body mass index between the carbohydrate and control groups or loss of fat mass from baseline to discharge.^21^ Nevertheless, preoperative oral glucose improved preservation of muscle mass compared with placebo. In contrast to the above findings, Mathur *et al* did not notice greater preservation of muscle mass in the carbohydrate group.^16^ Furthermore, carbohydrate treatment did not ameliorate postoperative nitrogen loss although it did increase the levels of insulin-like growth factor 1 postoperatively.

### Effect of preoperative carbohydrate in diabetic patients

Only one trial investigated the effect of carbohydrate drink in diabetic patients.^22^ The effect of carbohydrate drink was compared in 25 type 2 diabetic patients with 10 healthy controls. The gastric emptying rate was assessed using intestinal paracetamol absorption as a marker. Administration of carbohydrate drink 180 minutes before anaesthesia in uncomplicated diabetes patients is safe. It does not delay gastric emptying or cause hyperglycaemia.

## Discussion

The traditional practice of fasting patients before surgery results in depletion of hepatic glycogen, enhanced gluconeogenesis and insulin resistance.^23,24^ This is further aggravated by the insulin resistance caused by surgery.^25^ The practice of overnight fasting was first challenged in 1994 by Ljungqvist *et al* in patients undergoing an open cholecystectomy.^11^ Postoperative insulin resistance was reduced by 50% in patients receiving overnight intravenous glucose infusion. Moreover, in patients receiving glucose infusion, hepatic glycogen content was increased by 65% during surgery compared with fasting patients.^26^

Preoperative thirst has been suggested to be the main contributory factor of patient discomfort, followed by hunger and anxiety.^27^ Clear drinks alleviate thirst but their effect on hunger is inconclusive.^28,29^ Use of high carbohydrate drinks preoperatively was pioneered by Nygren *et al* in 1995.^12^ It was specially designed, consisting mainly of polymers to reduce the osmotic effect of the drink on gastric emptying. They demonstrated that the carbohydrate drink left the stomach in 90 minutes in patients undergoing a laparoscopic cholecystectomy after ingestion on the morning of surgery. None of the studies analysed in this review reported any adverse events following ingestion of carbohydrate drinks such as apparent or proven aspiration during or after surgery.

Preoperative carbohydrate drinks improved patient wellbeing after surgery significantly especially hunger thirst malaise anxiety and nausea. However, no benefit was noted by Bisgaard *et al* although the values were not statistically significant.^19^ A combination of heterogeneous surgical procedures, surgical access and anaesthetic protocols introduces a number of variables that could diminish the possibility of detecting any clinical benefit of carbohydrate drinks.^16^ A longer fasting time due to delay in the start of surgery and lower carbohydrate dose can also alleviate the effects of carbohydrate.

The review of trials examining the effect of carbohydrate on preservation of muscle mass presents a mixed picture and no conclusion could be drawn regarding the role of preoperative carbohydrate treatment. The varied methodology and outcome measures used could be a contributory factor. Future studies need to be carried out to investigate this further.

Factors that increase the risk of gastric aspiration are pregnancy, obesity, history of metabolic disorders including diabetes ASA grade >2 and gastrointestinal disorders. They were excluded from all the trials owing to fear of gastric aspiration resulting in pulmonary complications. No evidence was available with regard to the safety of use of carbohydrate drinks in these patients.

Diabetic patients are particularly at risk of poor glycaemic control after surgery.^30^ These patients have been excluded from the majority of the studies because of fear of delayed gastric emptying.^31,32^ In order to use preoperative carbohydrate loading in diabetic patients, it would be helpful to recognise patients with delayed gastric emptying. Since the correlation between gastric emptying rate and autonomic neuropathy and upper gastrointestinal symptoms is weak, physical examination and indirect tests are of little significance.^33–35^ The only study that examined the effects of carbohydrate drinks in diabetic patients was small (35 patients).^22^ The results cannot therefore be generalised to all diabetic patients. Furthermore, it needs to be explored whether carbohydrate loading has a similar beneficial effect on the metabolism as in non-diabetic patients.

Various oral carbohydrate preparations have been analysed and compared with placebo or overnight fasting (Table 6). The most commonly used oral formula for preoperative carbohydrate loading in the trials was a 12.5% carbohydrate drink (preOp® Nutricia, Trowbridge, UK) in quantities of 400ml or 800ml. It has been shown to be iso-osmolar and found to leave the stomach in 90 minutes with no reported adverse effects. The commercial preparation is available in a 200ml carton. The cost of one carton is £3.50 so it will cost £21 per patient (4 × 200ml in the evening before surgery and 2 × 200ml on the morning of the surgery) per procedure. One should evaluate whether this additional cost is worth the advantageous effects of the carbohydrate drink on clinical outcome.

**Table 6:** Costs of oral drinks used in various trials

Type of drink	Cost
preOp® (Nutricia, Trowbridge, UK)	£3.50 per 200ml (£21.00per patient per surgery)
Roosvicee Vruchtenmix (Heinz, Zeist, Netherlands) – syrup of rosehip and other fruits diluted in water, 70ml syrup: 330ml water	£3.99 per 200ml (£1.39 per patient per surgery)
100g Vitajoule® (Vitaflo, Liverpool, UK) dissolved in 800ml of water – pm, 50g Vitajoule® dissolved in 400ml of water – am	£3.77 per 500g (£1.13 per patient per surgery)
Aminoleban® EN (Otsuka Pharmaceutical, Tokyo, Japan) – mixture of carbohydrate and BCAAs, 100g per day given orally for 2 weeks	£13.00 per 450g (£40.00 per patient per surgery)

pm = evening before surgery; am = morning of surgery; BCAAs = branched chain amino acids

## Conclusions

Administration of oral carbohydrate drinks before surgery is probably safe as it leaves the stomach in 90 minutes and does not affect gastric acidity. It may have a positive influence on a wide range of perioperative markers of clinical outcome. There has been considerable focus in improving the recovery times and therefore shortening postoperative stay after both major and minor elective surgical procedures. This ethos has formed the basis of the enhanced recovery programme. Preoperative carbohydrate loading may be a useful adjunct in improving postoperative recovery. Further studies are required, however, to assess its cost effectiveness.
